# CXCL12 and eotaxin are independent prognostic serum biomarkers in gastric cancer

**DOI:** 10.1038/s41598-026-46511-z

**Published:** 2026-03-29

**Authors:** Jefim Brodkin, Tuomas Kaprio, Harri Mustonen, Alli Leppä, Arto Kokkola, Marko Salmi, Sirpa Jalkanen, Caj Haglund, Camilla Böckelman

**Affiliations:** 1 Translational Cancer Medicine Research Program, Faculty of Medicine, University of Helsinki, Haartmaninkatu 4, PO Box 340, 00029 Helsinki, Finland; 2 Department of Surgery, University of Helsinki and Helsinki University Hospital, Helsinki, Finland; 3 MediCity Research Laboratory, Institute of Biomedicine, University of Turku, Turku, Finland; 4 InFLAMES Flagship, University of Turku, Turku, Finland; 5 Department of Pathology, University of Helsinki and Helsinki University Hospital, Helsinki, Finland

**Keywords:** Gastric cancer, Survival, CXCL12, SCF, Eotaxin, Cancer, Molecular biology, Biomarkers, Gastroenterology

## Abstract

Gastric cancer is the fifth most common cancer and the fifth leading cause of cancer-related death worldwide. Its poor prognosis primarily results from a late diagnosis and the lack of effective treatments for advanced disease. Thus, we aimed to identify new prognostic serum biomarkers to aid clinical decision-making. Our patient cohort consisted of 240 individuals who underwent surgery for histologically verified gastric adenocarcinoma in the Department of Surgery at Helsinki University Hospital between 2000 and 2009. To determine the serum protein concentrations of cytokines and growth factors, we utilized Bio-Rad’s premixed Bio-Plex Pro Human Cytokine 27- and 21-plex assay kits. Among the 48 biomarkers we analyzed, three emerged as statistically significant prognostic markers for disease-specific survival using the Cox proportional hazards univariate analysis: C-X-C motif chemokine ligand 12 (CXCL12) (hazard ratio [HR] 0.39, 95% confidence interval [CI] 0.23–0.63, *p* < 0.001), stem cell factor (HR 0.38, 95% CI 0.19–0.77, *p* = 0.007), and eotaxin (HR 0.57, 95% CI 0.37–0.89, *p* = 0.013). Our multivariate survival analysis revealed that, among the 48 biomarkers analyzed, CXCL12 and eotaxin served as independent prognostic markers among gastric cancer patients. The prognostic effect of inflammatory serum biomarkers in gastric cancer may provide new insights into the immunological microenvironment of disease.

**Supplementary Information:**

The online version contains supplementary material available at 10.1038/s41598-026-46511-z.

## Introduction

Gastric cancer (GC) is a common cancer worldwide. Although its incidence has fallen in the Western world, GC remains one of the leading causes of cancer-related deaths, with the fifth highest incidence and fifth most common cause of cancer-related death globally^1^.

While the incidence of GC has decreased in Finland and other Western countries in recent decades, the prognosis for GC patients remains quite poor. The five-year survival rate in Finland from 2020 to 2022 was just above 30%^2^, similar to other Western countries that do not screen for GC^3^. Its poor prognosis primarily results from a late diagnosis and ineffective treatments for metastasized disease. To improve overall survival, new treatments and earlier diagnostics are needed.

Patient age and stage are known prognostic markers for GC. In addition, patients with a diffuse histology according to the Laurén classification^4^ exhibit a worse prognosis. Novel molecular subtypes such as those introduced by the Cancer Genome Atlas (TCGA)^5^ and the Asian Cancer Research Group (ACRG)^6^ may serve as potential prognostic markers.

Various serum biomarkers, such as C-reactive protein (CRP), carcinoembryonic antigen (CEA), and carbohydrate antigen 19 − 9 (CA19-9), are used in the diagnosis and follow-up of GC patients. However, their prognostic value remains unclear. For instance, Lu et al.^7^ found that patients with high pre-operative and/or post-operative CRP levels exhibited a worse prognosis. Other studies observed no or only a weak effect on survival^8,9^, although a meta-analysis detected an elevated CRP level in patients with a worse prognosis^10^. Yet Fent et al.^11^ found that patients with high CEA levels exhibited a worse survival, although CA19-9 did not serve as a prognostic factor. The levels of CEA and CA19-9 can also be used to assess the effect of neoadjuvant treatment, whereby normalization of their levels might indicate a better survival^12^. That said, GC patients with recurring disease and a worse prognosis had higher CEA levels, although no difference in the CA19-9 levels was observed^13^.

Cancer and inflammation are intertwined, such that GC is an example of an infection-driven cancer whereby most cases are associated with *Helicobacter pylori* (*H. pylori*) or Epstein–Barr virus (EBV) infection^14^. Chronic inflammation causes cancer, for instance, through the Correa pathway in GC^15^. Here, chronic gastritis, caused by *H. pylori*, leads to atrophic gastritis, and through a growing number of somatic mutations, progresses from intestinal metaplasia to dysplasia and eventually adenocarcinoma.

The most common subtype in the TCGA classification, chromosomal instability (CIN), is identified by mutated *TP53* and intestinal histology. The microsatellite instability (MSI) subtype reflects mutations to MSH2, MSH6, PMS2, or MLH1. The EBV-associated subtype is characterized by the *PIK3CA* mutation and *CDKN2A* silencing. The fourth subtype, genetically stable (GS), associates with a *CDH1* mutation and a diffuse histology, both of which are associated with the loss of cell-to-cell junctions and increased cellular motility. GC has been suggested as having immunological phenotypes according to the TCGA classification. For example, the CIN subtype exhibits less T-cell infiltration compared with the EBV subtype, and diffuse GC is associated with increased tertiary lymphoid structures^16^.

In this study, we utilized Bio-Rad’s premixed Bio-Plex Pro Human Cytokine 27- and 21-plex assay kits to identify possible serum biomarkers known to play a role in various cancers. We found that three serum biomarkers appeared to carry a prognostic value in GC: C-X-C motif chemokine ligand 12 (CXCL12), also known as stromal-derived factor 1 alpha (SDF-1α); stem cell factor (SCF), also known as the c-KIT ligand named after its receptor c-KIT; and eotaxin, also known as C-C motif chemokine 11 (CCL11).

## Results

### Univariate survival analysis

The multiplex analysis was successful for 29 biomarkers. However, for 18 biomarkers, more than 90% of the observed values fell below the standard curve and were omitted from further analysis. One biomarker, IL-3, yielded no results in the multiplex panel. The values for the serum concentrations of the 48 biomarkers were transformed to logarithm base 10 values.

Among the 29 biomarkers, we identified three which were statistically significant (*p* < 0.05) using the Cox proportional hazards univariate analysis: CXCL12 (hazard ratio [HR] 0.39, 95% confidence interval [CI] 0.23–0.63, *p* < 0.001, Table 1), SCF (HR 0.38, 95% CI 0.19–0.77, *p* = 0.007), and eotaxin (HR 0.57, 95% CI 0.37–0.89, *p* = 0.013). After a false discovery rate (FDR) correction, low levels of CXCL12 and SCF, respectively, significantly associated with a worse survival (*p* = 0.002 and 0.044, respectively, Supplementary Table 2).

### Multivariate survival analysis

In a multivariate analysis adjusted for median age, stage, the histological Laurén classification, the extent of the gastrectomy, adjuvant chemotherapy, adjuvant radiotherapy, and neoadjuvant therapy, CXCL12 and eotaxin emerged as statistically significant: HR 0.07 (95% CI 0.02–0.27, *p* < 0.001, C index 0.83 [standard error (SE) 0.02], Table 2a) for CXCL12, HR 0.27 (95% CI 0.07–1.05, *p* = 0.059, Table 2b) for SCF, and HR 0.40 (95% CI 0.18–0.89, *p* = 0.025, C index 0.82 [SE 0.02], Table 2c) for eotaxin.

**Table 1:** Univariate analysis of biomarkers using the Bio-Rad’s premixed Bio-Plex Pro Human Cytokine 21- and 27-plex assays.

	Median concentration (pg/ml)	IQR (pg/ml)	HR	95% CI	*p* value
CTACK	1340	977–1870	0.98	0.43–2.23	0.963
**CXCL12**	**1630**	**1340–2020**	**0.39**	**0.23–0.63**	**< 0.001**
**Eotaxin**	**205**	**147–284**	**0.57**	**0.37–0.89**	**0.013**
FGF-Basic	37.8	33.9–41.9	0.79	0.54–1.14	0.206
G-CSF	207	162–258	1.22	0.54–2.74	0.633
GM-CSF^a^	2.32	1.91–4.52	0.77	0.50–1.18	0.229
GRO*α*	264	241–293	0.73	0.36–1.45	0.369
HGF	811	621–1050	1.04	0.47–2.29	0.928
IFN-*α*2^a^	6.86	4.97–8.91	1.22	0.51–2.89	0.654
IFN-*γ*	15.2	11.4–21.2	0.95	0.51–1.77	0.872
IL-10^a^	7.41	5.64–13.7	1.17	0.66–2.08	0.600
IL-12(p40)^a^	88.0	61.3–117	0.86	0.48–1.56	0.631
IL-12(p70)^a^	4.56	2.00–8.78	1.16	0.76–1.77	0.487
IL-13	1.89	1.44–2.54	0.99	0.63–1.58	0.977
IL-15^a^	176	110–290	0.41	0.01–12.8	0.612
IL-16^a^	28.7	18.1–63.5	0.44	0.12–1.57	0.207
IL-17	8.57	6.71–10.5	1.41	0.93–2.15	0.110
IL-18	34.9	26.4–46.9	0.82	0.59–1.15	0.251
IL-1B	1.34	1.19–1.69	0.89	0.39–2.02	0.773
IL-1RA	179	135–228	1.00	1.00–1.00	0.067
IL-1*α*^a^	7.90	4.13–12.0	1.30	0.85–1.98	0.228
IL-2^a^	2.20	1.95–3.25	0.85	0.47–1.52	0.581
IL-2RA	74.4	59.0–96.1	0.69	0.29–1.63	0.393
IL-3	N/A	N/A	N/A	N/A	N/A
IL-4	1.50	1.20–1.80	0.58	0.28–1.21	0.146
IL-5^a^	21.7	18.5–76.2	2.02	0.29–14.2	0.481
IL-6^a^	1.96	1.19–4.35	0.78	0.55–1.11	0.170
IL-7	15.3	12.2–20.7	0.88	0.58–1.33	0.550
IL-8^a^	14.7	9.36–22.1	1.49	0.90–2.46	0.119
IL-9	275	252–294	0.51	0.23–1.15	0.105
IP-10	1739	1100–2810	1.44	0.92–2.23	0.130
LIF^a^	15.9	7.79–38.8	0.89	0.57–1.39	0.601
M-CSF	27.3	22.7–36.2	0.64	0.33–1.23	0.180
MCP-1	33.8	26.2–45.2	1.12	0.52–2.40	0.769
MCP-31	1.37	1.37–4.27	0.74	0.20–2.71	0.644
MIF	662	497–884	0.98	0.45–2.14	0.953
MIG^a^	1480	821–2730	1.06	0.69–1.63	0.774
MIP-1*α*	2.34	1.72–3.21	1.36	0.69–2.67	0.379
MIP-1*β*	422	391–459	0.48	0.14–1.60	0.233
PDGF-BB	4060	3100–5300	0.62	0.29–1.33	0.220
RANTES	13 400	11 800–15 300	1.07	0.33–3.46	0.916
**SCF**	**121**	**99.5–143**	**0.38**	**0.19–0.77**	**0.007**
SCGF-*β*	151 000	127 000–183 000	0.75	0.36–1.56	0.438
TNF-*α*	43.3	36.1–58.7	0.99	0.41–2.43	0.987
TNF-*β*1	56.0	26.4–109	0.48	0.12–1.94	0.301
TRAIL	124	91.9–146	1.00	0.36–2.74	0.995
VEGF^a^	237	118–439	0.73	0.34–1.54	0.410
*β*-NGF^a^	2.50	1.79–5.37	15.6	0.14–1717	0.253

^a^>90% of measurements fell below the standard curve.

IQR, interquartile range; HR, hazard ratio; CI, confidence interval.

All statistical analyses were calculated using the logarithmic values of the biomarkers.

**Table 2:** Multivariable Cox regression analysis for disease-specific survival.

	a			b			c		
	HR	95% CI	*p* value	HR	95% CI	*p* value	HR	95% CI	*p* value
Age									
<66	1.00			1.00			1.00		
≥66	2.13	1.39–3.26	**< 0.001**	2.24	1.45–3.47	**< 0.001**	2.10	1.38–3.20	**< 0.001**
Stage									
I	1.00			1.00			1.00		
II	7.24	2.42–21.6	**< 0.001**	7.79	2.62–23.2	**< 0.001**	8.30	2.78–24.7	**< 0.001**
III	27.3	9.57–77.8	**< 0.001**	27.4	9.64–78.2	**< 0.001**	27.8	9.73–79.2	**< 0.001**
IV	121	38.9–376	**< 0.001**	112	36.2–343	**< 0.001**	118	38.1–365	**< 0.001**
Laurén classification									
Intestinal	1.00			1.00			1.00		
Diffuse and mixed	2.31	1.52–3.51	**< 0.001**	2.04	1.35–3.09	**< 0.001**	2.05	1.34–3.14	**< 0.001**
Gastrectomy extent									
Distal gastrectomy	1.00			1.00			1.00		
Total gastrectomy	1.62	1.09–2.40	**0.017**	1.63	1.10–2.42	**0.015**	1.63	1.10–2.42	**< 0.001**
Adjuvant chemotherapy									
No	1.00			1.00			1.00		
Yes	0.57	0.34–0.98	**0.040**	0.60	0.35–1.01	0.052	0.67	0.39–1.13	0.130
Adjuvant radiotherapy									
No	1.00			1.00			1.00		
Yes	0.74	0.43–1.26	0.270	0.78	0.46–1.34	0.365	0.79	0.46–1.34	0.374
Neoadjuvant chemotherapy									
No	1.00			1.00			1.00		
Yes	2.06	1.03–4.10	**0.040**	2.24	1.13–4.46	**0.022**	2.17	1.09–4.31	**0.027**
**CXCL12**	0.07	0.02–0.27	**< 0.001**						
**SCF**				0.27	0.07–1.05	0.059			
**Eotaxin**							0.40	0.18–0.89	**0.025**

HR, hazard ratio; CI, confidence interval; CXCL12, C-X-C motif chemokine ligand 12; SCF, stem cell factor.

All statistical analyses were calculated using the logarithmic values of the biomarkers.

### Survival analysis of CXCL12, SCF, and eotaxin in patient subgroups

**Fig. 1:**
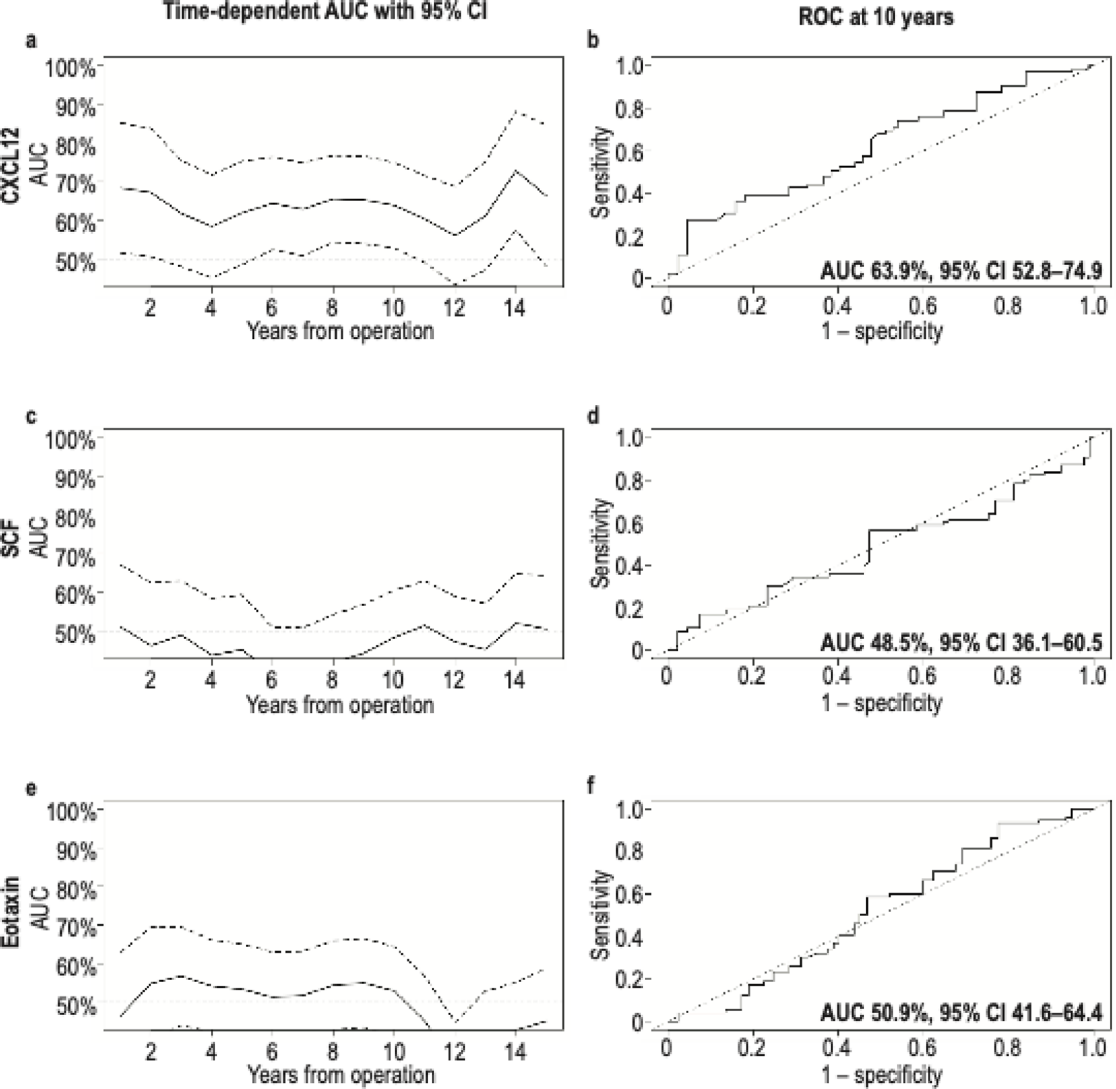
Time-dependent analysis of the area under the curve (AUC) and receiver operating characteristic (ROC) curve at the 10-year time point with 95% confidence intervals (CIs) for CXCL12 (**a** and** b**), SCF (**c** and** d**), and eotaxin (**e** and** f**), respectively. AUC, area under the curve; ROC, receiver operating characteristic; CI, confidence interval; CXCL12, C-X-C motif chemokine ligand 12; SCF, stem cell factor.

We analyzed the ability of three biomarkers to assess disease-specific survival (DSS) using the time-dependent area under the curve (AUC) values with 95% CIs (Fig. 1). Of the three biomarkers, CXCL12 was statistically significant at a majority of the time points (Fig. 1a). In the receiver operating characteristic (ROC) curve diagrams at the 10-year time point, CXCL12 had an AUC of 63.9% (95% CI 52.8–74.9). We then dichotomized the three biomarkers based on the maximum value of Youden’s index for the Kaplan–Meier analysis^17^.

**Fig. 2:**
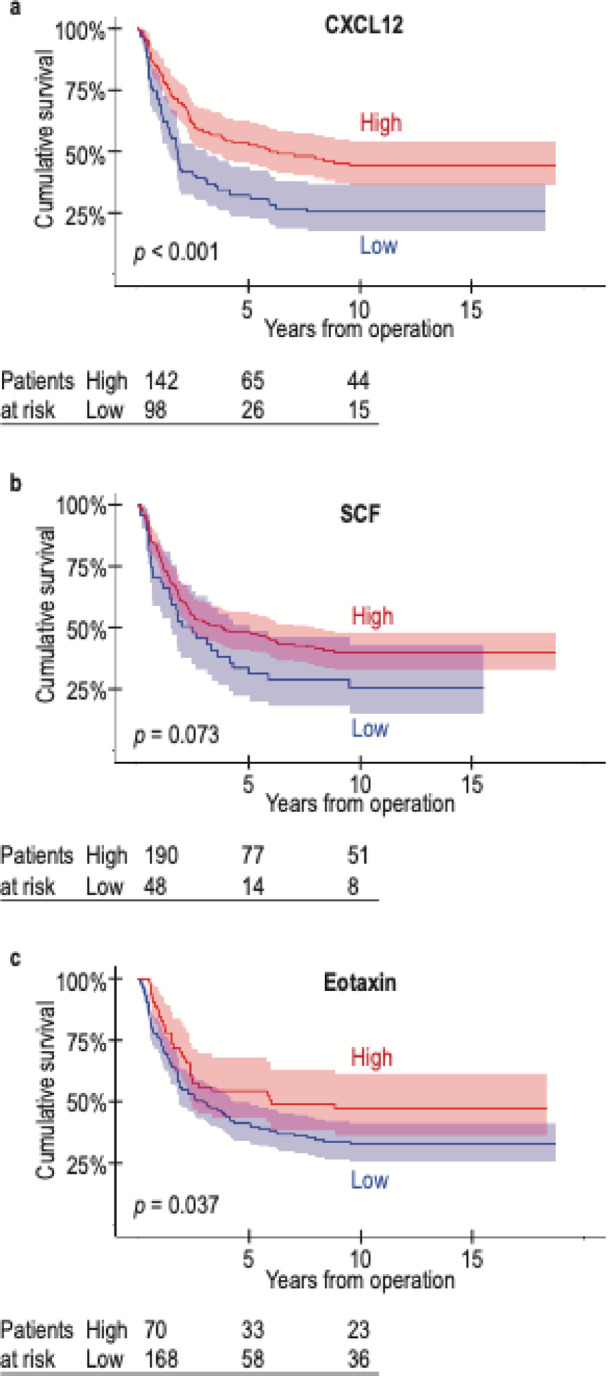
Disease-specific survival of patients with either a high or low serum concentration of CXCL12 (**a**), SCF (**b**), and eotaxin (**c**). Survival curves were drawn according to the Kaplan–Meier method and the *p* values were calculated using the log-rank test. CXCL12, C-X-C motif chemokine ligand 12; SCF, stem cell factor.

The estimated cutoff point for CXCL12 was 1513 pg/ml. The 5-year DSS among patients with high serum levels was 53.2% (95% CI 45.3–62.5), falling to 32.1% (95% CI 23.6–43.5, log rank test: *p* < 0.001, Fig. 2a) with low serum CXCL12 levels. The cutoff point for SCF was 97 pg/ml. The 5-year DSS among patients with high serum SCF levels was 48.1% (95% CI 41.2–56.2), falling to 33.6% (95% CI 22.1–51.0, *p* = 0.073, Fig. 2b) with low serum levels. The cutoff point for eotaxin was 267 pg/ml. The 5-year DSS among patients with high serum eotaxin levels was 54.4% (95% CI 43.4–68.2), falling to 41.3% (95% CI 34.2–49.9, *p* = 0.037, Fig. 2c) with low serum levels.

**Fig. 3:**
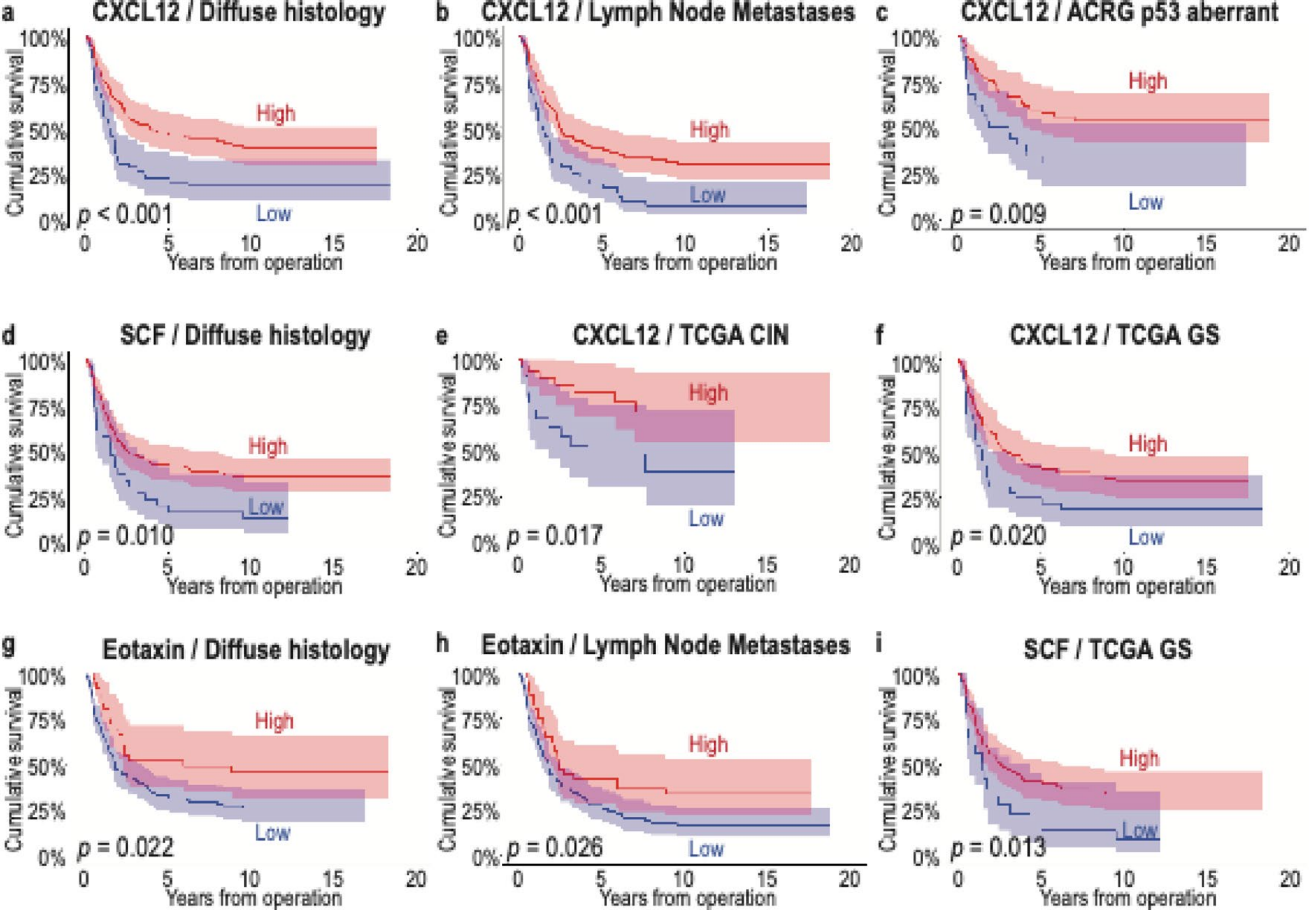
Disease-specific survival of patients with either a high or low serum concentration of CXCL12, SCF, and eotaxin in different patient subgroups. Patients with a diffuse histology (**a**), lymph node metastasis (**b**), or belonging to the p53 aberrant group in the ACRG classification (**c**), CIN (**e**) or GS (**f**) groups in the TCGA classification exhibited a better survival when CXCL12 levels were higher. Patients with a diffuse histology (**d**) or patients belonging to the GS group (**i**) of the TCGA classification experienced a better survival when the SCF levels were higher. In addition, in patients with high eotaxin levels, patients with a diffuse histology (**g**) or with lymph node metastasis (**h**) exhibited a better survival. Survival curves were drawn according to the Kaplan–Meier method and the *p* values were calculated using the log-rank test. ACRG, Asian Cancer Research Group; CIN, chromosomal instability; CXCL12, C-X-C motif chemokine ligand 12; GS, genetically stable; SCF, stem cell factor; TCGA, The Cancer Genome Atlas.

Among patients with a diffuse histology, the three serum biomarkers—CXCL12 (*p* < 0.001, Fig. 3a and Supplementary Tables 3 and 4), SCF (*p* = 0.010, Fig. 3d), and eotaxin (*p* = 0.022, Fig. 3g)—all served as prognostic factors. Among patients with lymph node metastases (pN+), those with higher levels of CXCL12 (*p* < 0.001, Fig. 3b) and eotaxin (*p* = 0.026, Fig. 3h) exhibited better survival.

We previously identified immunohistochemically determined prognostic patient subgroups using the TCGA and ACRG classifications^18^. Patients with high CXCL12 levels exhibited a better prognosis in the immunohistochemical ACRG classification’s p53 aberrant subtype (*p* = 0.009, Fig. 3c), with the TCGA classification’s subtype CIN characterized by an intestinal histology (*p* = 0.017, Fig. 3e) and genetically stable (GS) identified by a diffuse histology (*p* = 0.020, Fig. 3f). Similarly, patients with a high SCF concentration exhibited a better survival for the immunohistochemical TCGA classification’s GS subtype (*p* = 0.013, Fig. 3i).

### Associations between serum concentrations and clinicopathological variables

A high serum concentration of CXCL12 did not associate with the clinicopathological variables we examined (Table 3). However, a high serum concentration of SCF associated with an older age (*p* < 0.001) and stage of disease (*p* = 0.005), and a high serum concentration of eotaxin associated with distant metastasis (*p* = 0.046) and EBV positivity (*p* = 0.034).

## Discussion

Multivariate survival analysis revealed that, among the 48 biomarkers we analyzed, CXCL12 and eotaxin served as independent prognostic markers among gastric cancer (GC) patients. In addition, high levels of SCF also indicated a better survival. Subgroup analysis further revealed new associations between these novel serum biomarkers and other prognostic markers, similar to the Laurén classification and molecular subtypes described in our previous work^18^.

CXCL12 is a well-known cytokine associated with many pathologies and malignancies via signaling with its receptors CXCR4 and CXCR7^19^. In cancer, CXCL12 is involved in tumor progression, angiogenesis, metastasis, and survival through various downstream signaling pathways^19^. Contrary to other studies^20,21^, we found that a high level of CXCL12 served as an independent marker for a better prognosis in GC. Fewer studies, however, have examined the serum levels of CXCL12 than its expression in tumor tissue. Importantly, tumor and serum levels of CXCL12 are not necessarily comparable. CXCL12 can be expressed on the cell surface of both cancer cells as well as in the immune and other stromal cells^19^. Furthermore, CXCL12 can be secreted into the bloodstream, locally in the tissue, or simply expressed on the cell surface. Our results concerning the serum levels do not provide information on which cells have secreted the protein nor how it could be expressed in the tissue. A meta-analysis of CXCL12 expression showed that patients with high CXCL12 levels in the serum or tumor experienced a worse survival in esophagogastric and pancreatic cancer, whereas, in ovarian and colorectal cancer, the effect remained unclear^20^. In another study, gastric cancer patients with a high expression of CXCL12 in the tumor tissue did not exhibit a better survival^21^.

The AUC value of CXCL12 in our study was a moderate 63.9%, but was statistically significant at the majority of the time points we analyzed, suggesting its ability to effectively differentiate between groups. These modest AUC values represent a limitation to the applicability of our results. More recently, the role of CXCL12 in the tumor microenvironment (TME) and especially cancer-associated fibroblasts (CAFs) have been further explored. A higher CXCL12 expression associated with a pro-invasive inflammatory subset of CAFs, indicative of worse clinical outcomes^22^. In the subgroup analysis here, we observed that patients with a diffuse histology or lymph node metastasis experienced a better prognosis when they had higher serum levels of CXCL12. Previously, the serum and tumor levels of CXCL12 associated with lymph node and distant metastases of GC^23,24^. However, we found no association between elevated serum levels of CXCL12 and clinical or histological variables. Our results suggest that high CXCL12 levels in the serum are a systemic marker for a better survival.

**Table 3:** Associations between serum biomarker levels and clinicopathological variables.

	CXCL12^high^	*p* value^a^	SCF^high^	*p* value^a^	Eotaxin^high^	*p* value^a^
	*n* = 142 (59.2%)		*n* = 190 (79.8%)		*n* = 70 (29.5%)	
Age						
<66	75 (61.0)	0.559	87 (71.3)	**< 0.001**	37 (30.3)	0.783
≥66	67 (57.3)		103 (88.8)		33 (28.7)	
Sex						
Male	70 (59.8)	0.839	96 (82.1)	0.423	39 (33.3)	0.192
Female	72 (58.5)		94 (77.7)		31 (25.6)	
Stage						
I	36 (73.5)	0.078^b^	44 (89.8)	**0.005** ^**b**^	15 (30.6)	0.281^b^
II	29 (54.7)		46 (86.8)		17 (31.5)	
III	54 (56.3)		71 (75.5)		31 (33.3)	
IV	23 (54.8)		29 (69.0)		7 (16.7)	
Tumor invasion (pT)						
1	27 (75.0)	0.101^b^	32 (88.9)	0.066^b^	14 (38.9)	0.379^b^
2	22 (61.1)		32 (88.9)		10 (27.8)	
3	42 (52.5)		59 (74.7)		21 (26.3)	
4	51 (58.0)		67 (77.0)		25 (29.1)	
Lymph node metastasis (pN)						
No	47 (63.5)	0.444	63 (85.1)	0.165	25 (33.3)	0.384
Yes	92 (58.2)		120 (76.9)		43 (27.7)	
Distant metastasis (M)						
No	119 (60.1)	0.523	161 (82.1)	0.060	63 (32.1)	**0.046**
Yes	23 (54.8)		29 (69.0)		7 (16.7)	
Laurén classification						
Intestinal	47 (54.7)	0.288	68 (80.0)	0.962	31 (36.9)	0.061
Diffuse and other	95 (61.7)		122 (79.7)		39 (25.3)	
MMR						
MMRp	106 (61.6)	0.440	135 (78.9)	0.883	52 (30.4)	0.499
MMRd	22 (55.0)		32 (80.0)		10 (25.0)	
EBV *ish*						
EBV negative	127 (60.5)	0.908	166 (79.4)	0.577	58 (27.8)	**0.034**
EBV positive	5 (62.5)		7 (87.5)		5 (62.5)	
p53 staining						
Aberrant	105 (62.9)	0.230	134 (80.7)	0.350	48 (28.9)	0.908
Wild type	25 (53.2)		35 (74.5)		14 (29.8)	

^a^Pearson chi-square. ^b^Linear-by-linear association.

CXCL12, C-X-C motif chemokine ligand 12; SCF, stem cell factor; MMRp/d, mismatch repair proficient/deficient; EBVish, Epstein–Barr virus in situ hybridization.

The stem cell factor (SCF) is primarily expressed in hematopoietic cells, but can also be found in the gastric epithelium’s peristaltic pacemaker cells known as the interstitial cells of Cajal (ICCs)^25^. The expressions of SCF and its receptor c-KIT were previously identified in GC cells^26,27^, although their prognostic effect has not been established. We found that patients with high serum levels of SCF exhibited a better prognosis, although we did not establish a single cutoff point via which to categorize patients into distinct groups with a better and worse survival. The serum levels of SCF have been associated with GC in a multiplex setting alongside 18 other proteins as a proteomic tool for GC diagnosis, but not as an independent biomarker^28^. The c-KIT mutation can be found in different types of cancer, but one of the most studied examples of c-KIT’s involvement in cancer is the gastrointestinal stromal tumor (GIST), which originates from ICCs^25^. Patients with GIST have elevated tissue levels of SCF and advanced disease presumably by autocrine and paracrine activation of c-KIT signaling^29^. In another study, notably, the serum levels of SCF were lower in patients with GIST compared with healthy controls^30^. One study suggested that *H. pylori* infection causes the downregulation of SCF expression in gastric tissue, leading to fewer ICCs^31^. Moreover, SCF injections have been used as treatment for metastatic GC in a mouse model, where a subsequent increase in the number and activity of ICCs promoted normal peristaltic activity^32^. Low serum levels of SCF may indicate other non-beneficial effects on patients such as an impaired function of the gastrointestinal tract, whereas high serum levels promote a normal function. Interestingly, we found an association between high SCF serum levels and a lower stage of disease, yet identified no association with lymph node or distant metastasis.

Eotaxin, also known as C-C motif chemokine 11 (CCL11), is a chemokine primarily involved in guiding eosinophils, which affects allergic reactions^33^. Eotaxin and its receptor, chemokine receptor 3 (CCR3), are expressed by tumor cells and stromal cells in several different cancers exerting both pro- and anti-tumor effects^33^. We identified eotaxin as a novel independent prognostic biomarker in GC. While the prognostic effect of eotaxin was previously unclear, EBV-positive GC associated with elevated plasma levels of eotaxin, which we also confirmed^34^. That said, because only 5% of patients were EBV-positive, a larger patient cohort is necessary to validate these results. In another study, no associations between the eotaxin levels and clinicopathological variables were observed^35^. That said, elevated levels of eotaxin were noted in malignancies such as colorectal cancer, oral squamous cell cancer, and breast cancer^33^. Furthermore, eotaxin was previously identified as playing a role in angiogenesis and evading apoptosis, and may also play a part in GC^36,37^. However, the role of eotaxin in gastrointestinal malignancies is not yet well understood, warranting further research to elucidate the specific underlying biological mechanisms.

We previously identified prognostic serum biomarkers using the same multiplex panel of 48 serum biomarkers, exploring levels of cytokines and growth factors in colorectal cancer and pancreatic cancer^38,39^. In one previous study, the prognostic effect of serum biomarkers was explored, resulting in four candidates indicative of a worse survival in GC: IL-10Rb, adenosine deaminase (ADA), IL-20, and oncostatin M (OSM), which were not included in our panel of 48 cytokines^40^. In addition, multiplex analysis was used to identify diagnostic marker combinations for GC^41,42^. These findings, however, have not shed any further clarity on improving GC care. Thus, our preliminary results demand further validation before consideration for clinical use, preferably in different patient cohorts.

In our study, we used a rather large patient cohort of 240 patients comprised almost entirely of GC patients undergoing surgery at Helsinki University Hospital within a specific time frame. A larger sample with other patient cohorts would be needed to further validate our results given that we have conducted an exploratory study presenting novel findings. It is also worth noting that most serum samples were frozen at − 80 °C for more than ten years before an initial thawing for our analysis. It remains unclear how or if the proteins analyzed changed resulting from long-term storage. Some evidence suggests that long-term storage leads to the degradation of serum proteins^43^. However, our previous studies using samples stored for prolonged periods have been successful^38,39,44,45^. Furthermore, this was the first time the samples were thawed, likely the most crucial point in preserving samples for longer periods. Access to frozen samples has also allowed for a very long follow-up period exceeding ten years, allowing us the possibility to collect extensive data on survival. A considerable number of the biomarkers examined were omitted from further analysis because too many values fell outside the control values. Even though the higher number of excluded cytokines in this could result from technical limitations, according to our analysis, it is more likely that the serum levels were lower for some measured cytokines and growth factors. All of the analyses reported here were completed using kits from the same manufacturing lot, and we observed no correlation between values below the standard curve and the age of the samples. Other benefits to our sample included an older patient cohort resulting in the inclusion of almost no neoadjuvant-treated patients, making it possible to analyze comprehensive clinical data combined with histological samples without the potential influence of neoadjuvant chemotherapy. The primary strengths in this study are the relatively large patient cohort with a long follow-up period and the availability of comprehensive immunohistochemical data on patient samples.

Novel serum biomarkers could be used in addition to existing serum markers like CEA and CA19-9. Specifically, prognostic biomarkers could be used to identify patients with a better- or worse-than-average prognosis. Thus, identifying patients with a good prognosis could possibly avoid particularly intensive and exhausting treatments. Current treatment guidelines, for instance, do not recommend adjuvant chemotherapy for MSI-positive patients who already have a good prognosis since no additional benefit from adjuvant treatment has been observed^46^. Conversely, patients with a biomarker profile indicating a poor prognosis could be offered more aggressive treatments proactively. The effects of individual serum biomarkers were examined rather than creating a model that includes multiple biomarkers. The aim of this study was to identify individual biomarkers that could more easily be used in clinical settings. Although these biomarkers might not be suitable for individual use, they could be a part of a larger panel of biomarkers.

Both CXCL12 and eotaxin are chemokines primarily associated with inducing inflammation, further underlining the association between GC and infectious agents. Proinflammatory molecules may be indicative of a stronger immune response against tumors, perhaps partially explaining why patients with a higher systemic expression of these biomarkers experience a better prognosis. Our results point to the need to further examine the immunological landscapes of gastric cancer as possible new targets for prognostic evaluation and perhaps even treatment.

## Conclusions

The serum biomarkers CXCL12, SCF, and eotaxin can be used to assess prognosis in GC patients. The prognostic effect of inflammatory serum biomarkers CXCL12 and eotaxin might shed new light on our understanding of the immunological microenvironment of GC. Because our study was exploratory, we did not aim to examine the mechanistic explanations of our findings. Therefore, our results would benefit from further research and external validation. Additional investigation of these findings might yield mechanistic explanations, new treatment possibilities, and help better target treatments to specific patient subgroups.

## Materials and methods

### Patients

The patient cohort comprised 240 individuals who underwent surgery for histologically verified gastric adenocarcinoma in the Department of Surgery at Helsinki University Hospital between 2000 and 2009. The cohort consisted of patients who underwent both surgical treatment with a curative intent and who consented to participate in the study. However, we excluded patients with a history of malignant disease or synchronous cancer. There is no information regarding the presence of simultaneous serious inflammatory diseases at the time of the blood tests. The median age of the patient cohort at the time of surgery was 65.6 years (interquartile range [IQR] 56.5–75.5, Supplementary Table 1). Among all patients, 117 (48.8%) were male, and the median survival was 2.29 years (IQR 0.90–9.94). For staging, we used the seventh version of the tumor-node-metastasis (TNM) classification^47^. Overall, 49 (20.4%) had stage I cancer, 53 (22.1%) had stage II, 96 (40.0%) had stage III, and 42 (17.5%) had stage IV disease. An intestinal histology according to the Laurén classification was observed in 86 (35.8%) patients, while 154 (64.2%) had a diffuse or other type of histology. A distal gastrectomy was performed in 116 (48.3%) patients, while the remainder underwent a total gastrectomy. Most patients (*n* = 169, 71.3%) underwent at least a D2 lymphadenectomy, with others undergoing at least a D1 lymphadenectomy, and 98 (40.8%) patients undergoing a splenectomy. Adjuvant chemotherapy was administered to 100 (43.7%) patients, and 43 (19.1%) patients received adjuvant radiotherapy. Only 13 (5.4%) patients received neoadjuvant chemotherapy. Patients receiving adjuvant therapy were treated either with the MacDonald radiotherapy regimen and fluorouracil/leucovorin or with three rounds of epirubicin, oxaliplatin, and capecitabine (EOX).

### Serum samples

Serum samples were obtained from patients after possible neoadjuvant therapy one to two days before surgery, aliquoted, and subsequently stored at − 80 °C until the multiplex assay was performed in 2018.

### Protein profiling

To determine the serum protein concentrations of cytokines and growth factors, we used Bio-Rad’s premixed Bio-Plex Pro Human Cytokine 27-plex assay (catalog no. M500KCAF0Y) and 21-plex assay (catalog no. MF0005KMII) kits on Bio-Rad’s Bio-Plex 200 system (Supplementary Table 2). Assays were used according to the manufacturer’s instructions. However, only half of the recommended concentration levels of the detection antibodies, beads, and the streptavidin–phycoerythrin conjugate were used. We validated this approach in our previous studies^44,45^, and the approach was used successfully in other cancer patient cohorts^38,39^.

### Immunohistochemistry and determining the phenotypic subtypes

We constructed a tumor tissue microarray immunostained for the following markers: MSI markers MSH2, MSH6, MLH1, and PMS2; p53; E-cadherin; and EBER*ISH*. We used these stainings to divide patients into phenotypic subtypes according to the molecular subtypes of the TCGA and ACRG classifications^18^.

### Statistical analysis

We used two-tailed *p* values and considered *p* < 0.05 as statistically significant. Statistical evaluations were calculated using IBM’s statistical software (IBM SPSS Statistics Version 28, International Business Machines Corp., NY, USA) and R (R version 4.3.1, Foundation for Statistical Computing, Vienna, Austria). Associations between groups and continuous variables were assessed using the Mann–Whitney U-test and the Kruskal–Wallis test. For the univariate and multivariate analyses, we used the Cox proportional hazards regression analysis to calculate the disease-specific survival (DSS), and applied the false discovery rate (FDR) correction for multiple testing^48^. We defined DSS as the time from surgery until death from GC or until the end of the follow-up period. We chose patient characteristics consisting of age, stage (categorical variable), the Laurén classification, the extent of the gastrectomy, adjuvant chemotherapy, adjuvant radiotherapy, and neoadjuvant therapy for the multivariate survival analysis using the Cox regression model. We also tested for multicollinearity using the variance inflation factor (VIF) and accepted values < 5 as indicative of low collinearity. We calculated the time-dependent receiver operating characteristic (ROC) curves and the area under the curves (AUCs) using the TimeROC package in R, and the integrated AUC over time from 1 to 15 years. For the dichotomization of the biomarkers, we used the maximum value of Youden’s index at the 10-year time point^17^. For figures with Kaplan–Meier curves, statistical significance was calculated using the log-rank analysis.

## Supplementary Information

Below is the link to the electronic supplementary material.

Supplementary Material 1

## Data Availability

The datasets supporting the conclusions of this article are included within the article and its supplementary files. Other data used in this study are available from the corresponding author upon reasonable request.

## Abbreviations

ACRG: Asian Cancer Research Group
AUC: Area under the curve
CAF: Cancer-associated fibroblast
CA19-9: Carbohydrate antigen 19 − 9
CCL11: C–C motif chemokine 11
CEA: Carcinoembryonic antigen
CI: Confidence interval
CIN: Chromosomal instability
CRP: C-reactive protein
CXCL12: C-X-C motif chemokine ligand 12
DSS: Disease-specific survival
EBV: Epstein–Barr virus
FDR: False discovery rate
GC: Gastric cancer
GS: Genetically stable
HR: Hazard ratio
ICC: Interstitial cell of Cajal
IHC: Immunohistochemistry
IQR: Interquartile range
MMRp: Mismatch repair proficiency
OS: Overall survival
ROC: Receiver operating characteristic
SCF: Stem cell factor
SDF-1α: Stromal cell-derived factor 1 alpha
TCGA: The Cancer Genome Atlas
TME: Tumor microenvironment
TNM: Tumor-node-metastasis classification
