# Supplementary material for: CXCL12 and eotaxin are independent prognostic serum biomarkers in gastric cancer
Source: Sci Rep. 2026 Mar 29;16:10683. doi: 10.1038/s41598-026-46511-z (PMC13039986; doi:10.1038/s41598-026-46511-z)
Supplement: Supplementary file 1 — Supplementary Material 1 [file 41598_2026_46511_MOESM1_ESM.docx]

**Supplementary Table 1.** Patient characteristics

|  | n = 240 (%) |
| --- | --- |
| **Age** |  |
| <66 | 123 (51.2) |
| ≥66 | 117 (48.8) |
| **Sex** |  |
| Male | 117 (48.8) |
| Female | 123 (51.2) |
| **Stage** |  |
| I | 49 (20.4) |
| II | 53 (22.1) |
| III | 96 (40.0) |
| IV | 42 (17.5) |
| **Tumor invasion (pT)** |  |
| 1 | 36 (15.0) |
| 2 | 36 (15.0) |
| 3 | 80 (33.3) |
| 4 | 88 (36.7) |
| **Lymph node metastasis (pN)** |  |
| No | 74 (31.9) |
| Yes | 158 (68.1) |
| **Distant metastasis (M)** |  |
| No | 198 (82.5) |
| Yes | 42 (17.5) |
| **Laurén classification** |  |
| Intestinal | 86 (35.8) |
| Diffuse and other | 154 (64.2) |
| **Adjuvant chemotherapy** |  |
| No | 129 (56.3) |
| Yes | 100 (43.7) |
| **Adjuvant radiotherapy** |  |
| No | 182 (80.9) |
| Yes | 43 (19.1) |
| **Neoadjuvant chemotherapy** |  |
| No | 227 (94.6) |
| Yes | 13 (5.4) |
| **Gastrectomy extent** |  |
| Distal gastrectomy | 116 (48.3) |
| Total gastrectomy | 124 (51.7) |
| **Lymph node dissection** |  |
| D1 | 68 (28.7) |
| D2 or more | 169 (71.3) |
| **Splenectomy** |  |
| No | 142 (59.2) |
| Yes | 98 (40.8) |

**Supplementary Table 2.** Univariate analysis of biomarkers analyzed using Bio-Rad's premixed Bio-Plex Pro Human Cytokine 27- and 21-plex assays

|  |  | HR | 95% CI | *p* value | FDR-corrected *p* value |
| --- | --- | --- | --- | --- | --- |
| CTACK | Cutaneous T cell–attracting chemokine | 0.98 | 0.43–2.23 | 0.963 | 0.995 |
| CXCL12 | C-X-C motif chemokine ligand 12 | 0.39 | 0.23–0.63 | <0.001 | 0.002 |
| Eotaxin^b^ |  | 0.57 | 0.37–0.89 | 0.013 | 0.066 |
| FGF-Basic^b^ | Basic fibroblast growth factor | 0.79 | 0.54–1.14 | 0.206 | 0.494 |
| G-CSF^b^ | Granulocyte colony–stimulating factor | 1.22 | 0.54–2.74 | 0.633 | 0.876 |
| GM-CSF^a,b^ | Granulocyte-macrophage colony-stimulating factor | 0.77 | 0.50–1.18 | 0.229 |  |
| GRO𝛼 | Growth-regulated oncogene alpha | 0.73 | 0.36–1.45 | 0.369 | 0.673 |
| HGF | Hepatocyte growth factor | 1.04 | 0.47–2.29 | 0.928 | 0.995 |
| IFN-𝛼2^a^ | Interferon alpha 2 | 1.22 | 0.51–2.89 | 0.654 |  |
| IFN-𝛾^b^ | Interferon gamma | 0.95 | 0.51–1.77 | 0.872 | 0.995 |
| IL-10^a,b^ | Interleukin 10 | 1.17 | 0.66–2.08 | 0.600 |  |
| IL-12(p40)^a^ | Interleukin 12 (p40) | 0.86 | 0.48–1.56 | 0.631 |  |
| IL-12(p70)^a,b^ | Interleukin 12 (p70) | 1.16 | 0.76–1.77 | 0.487 |  |
| IL-13^b^ | Interleukin 13 | 0.99 | 0.63–1.58 | 0.977 | 0.995 |
| IL-15^a,b^ | Interleukin 15 | 0.41 | 0.01–12.8 | 0.612 |  |
| IL-16^a^ | Interleukin 16 | 0.44 | 0.12–1.57 | 0.207 |  |
| IL-17^b^ | Interleukin 17 | 1.41 | 0.93–2.15 | 0.110 | 0.395 |
| IL-18 | Interleukin 18 | 0.82 | 0.59–1.15 | 0.251 | 0.502 |
| IL-1RA^b^ | Interleukin 1 receptor antagonist | 1.00 | 1.00–1.00 | 0.067 | 0.303 |
| IL-1𝛼^a^ | Interleukin 1 alpha | 1.30 | 0.85–1.98 | 0.228 |  |
| IL-1𝛽^b^ | Interleukin 1 beta | 0.89 | 0.39–2.02 | 0.773 | 0.994 |
| IL-2^a,b^ | Interleukin 2 | 0.85 | 0.47–1.52 | 0.581 |  |
| IL-2RA | Interleukin 2 receptor antagonist | 0.69 | 0.29–1.63 | 0.393 | 0.673 |
| IL-4^b^ | Interleukin 4 | 0.58 | 0.28–1.21 | 0.146 | 0.439 |
| IL-5^a,b^ | Interleukin 5 | 2.02 | 0.29–14.2 | 0.481 |  |
| IL-6^a,b^ | Interleukin 6 | 0.78 | 0.55–1.11 | 0.170 |  |
| IL-7^b^ | Interleukin 7 | 0.88 | 0.58–1.33 | 0.550 | 0.826 |
| IL-8^a,b^ | Interleukin 8 | 1.49 | 0.90–2.46 | 0.119 |  |
| IL-9^b^ | Interleukin 9 | 0.51 | 0.23–1.15 | 0.105 | 0.395 |
| IP-10^b^ | Interferon gamma–induced protein 10 | 1.44 | 0.92–2.23 | 0.130 | 0.427 |
| LIF^a^ | Leukemia inhibitory factor | 0.89 | 0.57–1.39 | 0.601 |  |
| M-CSF | Macrophage colony-stimulating factor | 0.64 | 0.33–1.23 | 0.180 | 0.494 |
| MCP-1^b^ | Monocyte chemoattractant protein 1 | 1.12 | 0.52–2.40 | 0.769 | 0.994 |
| MCP-3^a^ | Monocyte chemoattractant protein 3 | 0.74 | 0.20–2.71 | 0.644 |  |
| MIF | Macrophage migration inhibitory factor | 0.98 | 0.45–2.14 | 0.953 | 0.995 |
| MIG^a^ | Monokine induced by gamma interferon | 1.06 | 0.69–1.63 | 0.774 |  |
| MIP-1𝛼^b^ | Macrophage inflammatory protein 1 alpha | 1.36 | 0.69–2.67 | 0.379 | 0.673 |
| MIP-1𝛽^b^ | Macrophage inflammatory protein 1 beta | 0.48 | 0.14–1.60 | 0.233 | 0.494 |
| PDGF-BB^b^ | Platelet-derived growth factor BB | 0.62 | 0.29–1.33 | 0.220 | 0.494 |
| RANTES^b^ | Regulated on activation, normal T cell expressed and secreted | 1.07 | 0.33–3.46 | 0.916 | 0.995 |
| SCF | Stem cell factor | 0.38 | 0.19–0.77 | 0.007 | 0.044 |
| SCGF-𝛽 | Serum stem cell growth factor beta | 0.75 | 0.36–1.56 | 0.438 | 0.716 |
| TNF-𝛼^b^ | Tumor necrosis factor alpha | 0.99 | 0.41–2.43 | 0.987 | 0.995 |
| TNF-𝛽^a^ | Tumor necrosis factor beta | 0.48 | 0.12–1.94 | 0.301 |  |
| TRAIL | TNF-related apoptosis-inducing ligand | 1.00 | 0.36–2.74 | 0.995 | 0.995 |
| VEGF^a,b^ | Vascular endothelial growth factor | 0.73 | 0.34–1.54 | 0.410 |  |
| 𝛽-NGF^a^ | Nerve growth factor beta | 15.6 | 0.14–1717 | 0.253 |  |
| ^a^Measurements fell below the standard curve. | |  |  |  |  |
| ^b^Cytokines and growth factors from Bio-Rad’s premixed Bio-Plex Pro Human Cytokine 27- plex assay. | | | |  |  |
| Abbreviations: IQR, interquartile range; HR, hazard ratio; CI, confidence interval; FDR, false discovery rate. | | | | |  |
| All statistical analyses were completed using the logarithmic values of the biomarkers as continuous variables. | | |  |  |  |

**Supplementary Table 3.** Univariate survival analysis of dichotomized serum biomarkers where low serum levels serve as the reference value (HR = 1.00)

|  | **CXCL12** | | | **SCF** | | | **Eotaxin** | | |
| --- | --- | --- | --- | --- | --- | --- | --- | --- | --- |
|  | HR | 95% CI | *p* value | HR | 95% CI | *p* value | HR | 95% CI | *p* value |
| **Age** |  |  |  |  |  |  |  |  |  |
| <66 | 0.44 | 0.27–0.70 | **<0.001** | 0.42 | 0.26–0.69 | **<0.001** | 0.63 | 0.36–1.08 | 0.094 |
| ≥66 | 0.74 | 0.46–1.20 | 0.216 | 1.60 | 0.69–3.70 | 0.277 | 0.72 | 0.41–1.26 | 0.244 |
| **Sex** |  |  |  |  |  |  |  |  |  |
| Male | 0.64 | 0.39–1.06 | 0.085 | 0.73 | 0.39–1.34 | 0.309 | 0.82 | 0.48–1.40 | 0.474 |
| Female | 0.49 | 0.31–0.78 | **0.002** | 0.69 | 0.41–1.15 | 0.151 | 0.52 | 0.29–0.95 | **0.034** |
| **Stage** |  |  |  |  |  |  |  |  |  |
| I | 1.14 | 0.12–11.0 | 0.910 | N/A |  |  | 0.81 | 0.08–7.78 | 0.855 |
| II | 0.55 | 0.23–1.31 | 0.176 | 1.32 | 0.31–5.69 | 0.711 | 0.39 | 0.13–1.15 | 0.088 |
| III | 0.46 | 0.29–0.73 | **<0.001** | 1.04 | 0.62–1.76 | 0.878 | 0.86 | 0.52–1.41 | 0.541 |
| IV | 1.00 | 0.53–1.92 | 0.989 | 0.91 | 0.45–1.81 | 0.781 | 0.40 | 0.16–1.04 | 0.061 |
| **Tumor invasion (pT)** |  |  |  |  |  |  |  |  |  |
| 1 | 0.16 | 0.02–1.78 | 0.137 | N/A |  |  | 0.79 | 0.07–8.69 | 0.845 |
| 2 | 0.89 | 0.26–3.01 | 0.855 | 0.52 | 0.11–2.43 | 0.409 | 0.86 | 0.23–3.25 | 0.821 |
| 3 | 0.56 | 0.33–0.96 | **0.034** | 1.05 | 0.56–1.97 | 0.871 | 0.81 | 0.43–1.50 | 0.497 |
| 4 | 0.65 | 0.41–1.05 | 0.075 | 0.76 | 0.44–1.30 | 0.307 | 0.53 | 0.30–0.93 | **0.027** |
| **Lymph node metastasis (pN)** |  |  |  |  |  |  |  |  |  |
| No | 0.59 | 0.23–1.54 | 0.281 | 0.55 | 0.18–1.69 | 0.297 | 0.82 | 0.29–2.31 | 0.712 |
| Yes | 0.51 | 0.35–0.74 | **<0.001** | 0.79 | 0.52–1.22 | 0.289 | 0.61 | 0.39–0.95 | **0.027** |
| **Distant metastasis (M)** |  |  |  |  |  |  |  |  |  |
| No | 0.48 | 0.32–0.71 | **<0.001** | 0.77 | 0.47–1.24 | 0.278 | 0.80 | 0.52–1.24 | 0.324 |
| Yes | 1.00 | 0.53–1.92 | 0.989 | 0.91 | 0.45–1.81 | 0.781 | 0.40 | 0.16–1.04 | 0.061 |
| **Laurén classification** |  |  |  |  |  |  |  |  |  |
| Intestinal | 0.54 | 0.29–1.02 | 0.058 | 1.18 | 0.52–2.67 | 0.700 | 1.01 | 0.52–1.96 | 0.981 |
| Diffuse and other | 0.51 | 0.34–0.76 | **<0.001** | 0.56 | 0.38–0.88 | **0.012** | 0.56 | 0.34–0.93 | **0.024** |
| **MMR** |  |  |  |  |  |  |  |  |  |
| MMRp | 0.56 | 0.38–0.83 | **0.004** | 0.67 | 0.43–1.06 | 0.085 | 0.73 | 0.46–1.14 | 0.163 |
| MMRd | 0.77 | 0.36–1.67 | 0.508 | 0.95 | 0.38–2.37 | 0.911 | 0.43 | 0.15–1.24 | 0.119 |
| **EBV *ish*** |  |  |  |  |  |  |  |  |  |
| EBV negative | 0.56 | 0.39–0.80 | **0.001** | 0.68 | 0.45–1.03 | 0.071 | 0.65 | 0.42–1.00 | **0.047** |
| EBV positive | 0.61 | 0.12–3.08 | 0.548 | 1.28 | 0.15–11.3 | 0.823 | 0.49 | 0.10–2.44 | 0.382 |
| **p53 staining** |  |  |  |  |  |  |  |  |  |
| Aberrant | 0.52 | 0.35–0.79 | **0.002** | 0.70 | 0.43–1.14 | 0.151 | 0.65 | 0.41–1.04 | 0.074 |
| Wild type | 0.75 | 0.37–1.53 | 0.430 | 0.73 | 0.34–1.56 | 0.421 | 0.77 | 0.33–1.80 | 0.550 |
| **ACRG** |  |  |  |  |  |  |  |  |  |
| p53aber | 0.49 | 0.28–0.85 | **0.011** | 0.57 | 0.31–1.04 | 0.065 | 0.53 | 0.27–1.04 | 0.063 |
| p53wt | 1.04 | 0.37–2.86 | 0.948 | 0.82 | 0.28–2.40 | 0.713 | 1.23 | 0.035–4.37 | 0.752 |
| MSI | 0.77 | 0.36–1.67 | 0.508 | 0.95 | 0.38–2.37 | 0.911 | 0.43 | 0.15–1.24 | 0.119 |
| EMT | 0.51 | 0.25–1.01 | 0.053 | 0.61 | 0.24–1.60 | 0.317 | 0.92 | 0.46–1.85 | 0.815 |
| **TCGA** |  |  |  |  |  |  |  |  |  |
| CIN | 0.34 | 0.13–0.86 | **0.023** | 1.06 | 0.35–3.19 | 0.922 | 1.25 | 0.48–3.23 | 0.644 |
| GS | 0.58 | 0.36–0.92 | **0.021** | 0.53 | 0.31–0.09 | **0.014** | 0.64 | 0.37–1.11 | 0.109 |
| MSI | 0.77 | 0.36–1.67 | 0.508 | 0.95 | 0.38–2.37 | 0.911 | 0.43 | 0.15–1.24 | 0.119 |
| EBV | 0.61 | 0.12–3.08 | 0.504 | 1.28 | 0.15–11.3 | 0.823 | 0.49 | 0.10–2.44 | 0.382 |
| Abbreviations: CXCL12, C-X-C motif chemokine ligand 12; SCF, stem cell factor; HR, hazard ratio; | | | | | | | | | |
| CI, confidence interval; MMRp/d, mismatch repair proficient/deficient; EBVish, Epstein–Barr virus *in situ* | | | | | | | | | |
| hybridization; ACRG, Asian Cancer Research Group; p53aber/wt, p53 aberrant/wild-type; MSI, | | | | | | | | | |
| microsatellite instability; EMT, epithelial–mesenchymal transition; TCGA, The Cancer Genome Atlas; | | | | | | | | | |
| CIN, chromosomal instability; GS, genetically stable. | | | | |  |  |  |  |  |

**Supplementary Table 4**. Univariate analysis of biomarkers as continuous variables in patient subgroups

|  | **CXCL12** | | | **SCF** | | | **Eotaxin** | | |
| --- | --- | --- | --- | --- | --- | --- | --- | --- | --- |
|  | HR | 95% CI | *p* value | HR | 95% CI | *p* value | HR | 95% CI | *p* value |
| **Age** |  |  |  |  |  |  |  |  |  |
| <66 | 0.08 | 0.02–0.40 | **0.002** | 0.11 | 0.03–0.43 | **0.002** | 0.47 | 0.16–1.4 | 0.169 |
| ≥66 | 0.56 | 0.06–5.8 | 0.629 | 1.5 | 0.28–8.4 | 0.628 | 0.93 | 0.33–2.6 | 0.890 |
| **Sex** |  |  |  |  |  |  |  |  |  |
| Male | 0.23 | 0.02–2.5 | 0.223 | 0.35 | 0.05–2.4 | 0.291 | 0.59 | 0.17–2.0 | 0.400 |
| Female | 0.14 | 0.03–0.65 | **0.013** | 0.519 | 0.12–2.2 | 0.374 | 0.72 | 0.27–1.9 | 0.500 |
| **Stage** |  |  |  |  |  |  |  |  |  |
| I | 1.92 | 0.00–46000 | 0.899 | 11 | 0.45–290 | 0.141 | 1.69 | 0.02–120 | 0.809 |
| II | 0.35 | 0.00–37 | 0.657 | 0.59 | 0.02–23 | 0.779 | 0.46 | 0.04–4.9 | 0.516 |
| III | 0.14 | 0.03–0.61 | **0.009** | 0.69 | 0.15–3.1 | 0.624 | 0.57 | 0.21–1.59 | 0.283 |
| IV | 2.18 | 0.13–38 | 0.591 | 0.76 | 0.04–14 | 0.854 | 0.25 | 0.06–0.99 | **0.049** |
| **Tumor invasion (pT)** | |  |  |  |  |  |  |  |  |
| 1 | 0.00 | 0.00–3.7 | 0.071 | 12 | 0.41–360 | 0.150 | 2.5 | 0.03–230 | 0.696 |
| 2 | 0.50 | 0.00–260 | 0.829 | 0.15 | 0.00–44 | 0.512 | 0.47 | 0.02–13 | 0.650 |
| 3 | 0.22 | 0.04–1.3 | 0.095 | 0.40 | 0.09–1.9 | 0.241 | 0.61 | 0.19–2.0 | 0.419 |
| 4 | 0.27 | 0.03–3.0 | 0.287 | 0.56 | 0.08–4.2 | 0.578 | 0.15 | 0.04–0.49 | **0.002** |
| **Lymph node metastasis (pN)** | | |  |  |  |  |  |  |  |
| No | 0.14 | 0.00–17 | 0.423 | 1.0 | 0.06–19 | 0.984 | 1.3 | 0.15–11 | 0.830 |
| Yes | 0.22 | 0.05–0.90 | **0.034** | 0.43 | 0.12–1.5 | 0.189 | 0.38 | 0.17–0.85 | **0.019** |
| **Distant metastasis (M)** | |  |  |  |  |  |  |  |  |
| No | 0.08 | 0.02–0.35 | **<0.001** | 0.59 | 0.15–2.3 | 0.438 | 0.99 | 0.40–2.5 | 0.984 |
| Yes | 2.2 | 0.13–38 | 0.591 | 0.76 | 0.04–14 | 0.854 | 0.25 | 0.06–0.99 | **0.049** |
| **Laurén classification** | |  |  |  |  |  |  |  |  |
| Intestinal | 0.25 | 0.01–6.7 | 0.409 | 1.4 | 0.18–11 | 0.744 | 1.8 | 0.50–6.8 | 0.365 |
| Diffuse and other | 0.12 | 0.03–0.47 | **0.002** | 0.35 | 0.09–1.3 | 0.116 | 0.40 | 0.16–0.98 | **0.044** |
| **Adjuvant chemotherapy** | |  |  |  |  |  |  |  |  |
| No | 0.04 | 0.00–0.50 | **0.013** | 0.95 | 0.17–5.3 | 0.951 | 1.0 | 0.29–3.5 | 0.985 |
| Yes | 0.24 | 0.04–1.4 | 0.110 | 0.30 | 0.07–1.4 | 0.132 | 0.26 | 0.10–0.69 | **0.007** |
| **Adjuvant radiotherapy** | |  |  |  |  |  |  |  |  |
| No | 0.06 | 0.01–0.44 | **0.005** | 0.41 | 0.10–1.6 | 0.198 | 0.85 | 0.34–2.1 | 0.715 |
| Yes | 0.11 | 0.01–1.3 | 0.080 | 0.27 | 0.02–3.6 | 0.325 | 0.24 | 0.05–1-2 | 0.079 |
| **Neoadjuvant chemotherapy** | |  |  |  |  |  |  |  |  |
| No | 0.16 | 0.04–0.61 | **0.008** | 0.40 | 0.12–1.3 | 0.131 | 0.67 | 0.31–1.5 | 0.313 |
| Yes | 0.42 | 0.00–870 | 0.825 | 2.1 | 0.02–220 | 0.752 | 0.19 | 0.01–5.1 | 0.325 |
| **Gastrectomy extent** |  |  |  |  |  |  |  |  |  |
| Distal gastrectomy | 0.29 | 0.02–4.6 | 0.378 | 0.30 | 0.04–2.3 | 0.246 | 0.71 | 0.21–2.4 | 0.581 |
| Total gastrectomy | 0.17 | 0.04–0.69 | **0.014** | 0.71 | 0.17–3.0 | 0.645 | 0.51 | 0.20–1-3 | 0.170 |
| **Lymph node dissection** | |  |  |  |  |  |  |  |  |
| D1 | 0.20 | 0.02–2.6 | 0.220 | 0.13 | 0.02–1.1 | 0.056 | 0.68 | 0.15–3.0 | 0.607 |
| D2 or more | 0.13 | 0.03–0.67 | **0.015** | 0.54 | 0.13–2.3 | 0.412 | 0.88 | 0.35–2.3 | 0.796 |
| **TCGA** |  |  |  |  |  |  |  |  |  |
| CIN | 0.14 | 0.00–14 | 0.409 | 1.1 | 0.08–15 | 0.960 | 3.6 | 0.60–22 | 0.163 |
| GS | 0.20 | 0.04–0.98 | **0.048** | 0.25 | 0.07–0.93 | **0.039** | 0.58 | 0.21–1.6 | 0.306 |
| MSI | 0.97 | 0.02–58 | 0.989 | 5.8 | 0.21–160 | 0.302 | 0.12 | 0.02–0.69 | **0.017** |
| EBV | 1.7 | 0.00–37000 | 0.916 | 0.03 | 0.00–130 | 0.424 | 0.04 | 0.00–3.2 | 0.150 |
| **ACRG** |  |  |  |  |  |  |  |  |  |
| p53aberrant | 0.18 | 0.01–2.5 | 0.201 | 0.14 | 0.02–1.1 | 0.063 | 0.76 | 0.22–2.6 | 0.669 |
| p35wild-type | 1.9 | 0.01–580 | 0.830 | 0.32 | 0.00–26 | 0.615 | 5.7 | 0.34–96 | 0.225 |
| MSI | 0.97 | 0.02–58 | 0.989 | 5.8 | 0.21–160 | 0.302 | 0.12 | 0.02–0.69 | **0.017** |
| EMT | 0.32 | 0.05–2.3 | 0.253 | 0.47 | 0.11–2.0 | 0.315 | 1.0 | 0.26–4.0 | 0.976 |
| Abbreviations: CXCL12, C-X-C motif chemokine ligand 12; SCF, stem cell factor; HR, hazard ratio; CI, confidence interval; EBV, Epstein–Barr virus; ACRG, Asian Cancer Research Group; MSI, microsatellite instability; EMT, epithelial–mesenchymal transition; TCGA, The Cancer Genome Atlas; CIN, chromosomal instability; GS, genetically stable. | | | | | | |  |  |  |
